# The way you say it, the way I feel it: emotional word processing in accented speech

**DOI:** 10.3389/fpsyg.2015.00351

**Published:** 2015-03-27

**Authors:** Anna Hatzidaki, Cristina Baus, Albert Costa

**Affiliations:** ^1^Center of Brain and Cognition, Universitat Pompeu FabraBarcelona, Spain; ^2^Laboratoire de Psychologie Cognitive, Centre National de la Recherche Scientifique - Université Aix-MarseilleMarseille, France; ^3^Institució Catalana de Recerca i Estudis AvançatsBarcelona, Spain

**Keywords:** emotion, affective valence, native and foreign accent, spoken word processing, event-related potentials

## Abstract

The present study examined whether processing words with affective connotations in a listener's native language may be modulated by accented speech. To address this question, we used the Event Related Potential (ERP) technique and recorded the cerebral activity of Spanish native listeners, who performed a semantic categorization task, while listening to positive, negative and neutral words produced in standard Spanish or in four foreign accents. The behavioral results yielded longer latencies for emotional than for neutral words in both native and foreign-accented speech, with no difference between positive and negative words. The electrophysiological results replicated previous findings from the emotional language literature, with the amplitude of the Late Positive Complex (LPC), associated with emotional language processing, being larger (more positive) for emotional than for neutral words at posterior scalp sites. Interestingly, foreign-accented speech was found to interfere with the processing of positive valence and go along with a negativity bias, possibly suggesting heightened attention to negative words. The manipulation employed in the present study provides an interesting perspective on the effects of accented speech on processing affective-laden information. It shows that higher order semantic processes that involve emotion-related aspects are sensitive to a speaker's accent.

## Introduction

Conversations in which interlocutors have different accents are not uncommon. Differences in accent may come from different dialectal or social variations, as well as from variations due to using a foreign language. Given the globalized world, interacting with people that have a foreign accent is thus a frequent phenomenon. The pervasiveness of this communicative situation calls for a better understanding of the cognitive and linguistic processes that are at play when we process foreign-accented speech.

There is substantial evidence showing pervasive effects of foreign accent in many cognitive and social contexts. For example, adults tend to judge more positively (in terms of social status, education, professional success, and credibility) speakers with a native than with a foreign accent (Ryan, 1983; Giles and Billings, 2004; Lev-Ari and Keysar, 2010; Fuertes et al., 2012; Pantos and Perkins, 2013). Children, monolingual and bilingual alike, have also been found to base part of their social preferences on accent, showing a decided preference to befriend native-accented peers over foreign-accented peers (Kinzler et al., 2009; Souza et al., 2013).

More important for our purposes, however, is the fact that some linguistic processes seem to be also affected by the presence of foreign accent. Behavioral studies have showed, for example, that under degraded listening conditions, listeners of foreign-accented speech make use of lexical information in a strategic manner to facilitate phonemic category decisions (Bürki-Cohen et al., 2001), or that under normal listening conditions, foreign accent can modulate listeners' expectations of appropriate word pronunciation (Schmid and Yeni-Komshian, 1999). Moreover, it has been suggested that foreign accent may create the anticipation of a less native-like language performance and by extension drive native listeners into constructing less detailed discourse representations (Lev-Ari and Keysar, 2012) and shallower semantic processing (Goslin et al., 2012; but see Hanulíková et al., 2012; Romero-Rivas et al., 2015), or into showing more tolerance toward ungrammatical foreign-accented speech than toward ungrammatical native-accented speech (Hanulíková et al., 2012).

Although the above-mentioned effects are not trivial, only a handful of studies have examined the interplay of foreign accent and linguistic processing, with electrophysiological evidence—that would allow a finer picture of the phenomenon—being scarce (see Goslin et al., 2012 for a short discussion). Yet the use of EEG, especially that of Event-Related Potentials (ERPs), is the appropriate instrument to detect and examine foreign accent effects. Unlike behavioral measures, electrophysiological measures allow a more informative description of cognitive functions that may not be captured by less sensitive measures. In ERPs, brain activity is time-locked to the presentation of a given stimulus and provides information about its processing (in terms of time, polarity, and distribution of effects) through associated ERP components (see Kutas and Van Petten, 1988; Osterhout et al., 1997 for reviews). Clearly, such an approach can provide insights about how basic linguistic processes are modulated by foreign accent and help us also understand the communicative problems that are often encountered when interacting with foreign-accented speakers.

## The present study

Aiming to fill these gaps, the present study took research on foreign accent effects one step further, bridging the research areas of social, cognitive, and language sciences. Specifically, it focused on processes related to emotional reaction elicited by affective-laden words and, using the ERP technique, explored if semantic processing of emotional words is influenced to some extent by whether those words are spoken with a foreign accent.

The case of emotional words is genuinely interesting, because words with affective connotations, apart from carrying lexical-semantic information, additionally activate the limbic system, particularly the amygdala, which allows access to information that generates emotional experiences (e.g., Straube et al., 2011; Keuper et al., 2014). Take for example the concept REGALO (“gift” in Spanish). It arguably contains more positive associations than the concept LAVABO (“sink” in Spanish). Thus, the former is considered an emotionally positive word (Redondo et al., 2007), because a *regalo* is usually given or received to mark a happy occasion or to express kind feelings, such as gratitude and appreciation. Hence, processing an emotional word's referent typically leads to emotion generation (Lang, 1979; Bower, 1981). *Lavabo*, on the other hand, is considered a neutral word, because it is used for mundane purposes of daily life and is not particularly associated with positive or negative connotations.

If accented speech can impact linguistic and higher order processes required for processing affective-laden information, then we would expect an emotional word such as *regalo* to be sensitive to the effects of foreign-accented speech. Such a finding could have important implications, not only for interpersonal communication, but also for models of word representation and word processing in general and of emotional word processing in particular. It would suggest that semantic and emotion-related content processing can be modulated by whether the phonological realization of an emotional word is familiar to its listener or not. Crucially, it would also suggest that accent may influence deep rooted thoughts and human experiences related to emotion.

To examine whether foreign-accented speech can indeed impact the linguistic processing of affective-laden words, we employed the ERP methodology, which has been used extensively in the emotional language literature, predominantly in the visual modality. Electrophysiological studies that have used ERPs to examine how language processes can be affected by foreign accent have yielded mixed findings, most likely reflecting the different processes encouraged by the tasks employed (e.g., semantic categorization vs. semantic or syntactic violation), as well as the differential role of involvement of sentential context (Goslin et al., 2012; Hanulíková et al., 2012; Romero-Rivas et al., 2015). Reported effects from these studies and relevant ERP components (Phonological Mapping Negativity, N400, P600) suggest that foreign accent may not be sufficiently normalized at early stages of word processing and as a result exert its effects during meaning integration (Goslin et al., 2012, but see Hanulíková et al., 2012). The conditions under which this occurs may be also subject to foreign accent adaptation (Romero-Rivas et al., 2015).

Effects of emotional word processing at an electrophysiological level are clearer and more consistent: they are typically shown in the modulation of the Late Positive Complex (LPC), which—depending on the task at hand and the experimental manipulations—may be preceded by effects on earlier components (e.g., N1, P2, EPN, N400). The LPC, also reported as LPP (Late Positive Potential), is an ERP modality independent component associated with emotional language processing (see Kotz and Paulmann, 2011; Citron, 2012 for reviews). Modulation of the amplitude of the LPC usually shows at around 500 ms post-stimulus onset, lasts hundreds of milliseconds, and has a centro-parietal locus. It is argued to reflect sustained attention to emotional input, and is found, for instance, when contrasting positive or negative valence words to neutral words, with a larger (more positive) amplitude for emotional than for neutral stimuli (e.g., Frühholz et al., 2011; Kissler and Herbert, 2012).

To our knowledge, there are only two ERP studies (both coming from the same laboratory) that have looked at the processing of auditory, emotion-related single words (Mittermeier et al., 2011; Jaspers-Fayer et al., 2012). In Mittermeier et al. (2011) the focus was on the identification of an ERP component that would also relate to the personality dimension of extroversion-introversion. The participants were native listeners of German and had to perform three tasks in a row: tone discrimination (high vs. low; control condition); affective-syllable intonation discrimination (positive vs. negative; affective valence condition 1); and affective-word meaning discrimination (positive vs. negative, produced in a “monotonous female voice,” affective valence condition 2). The analyses involved the comparison of behavioral and electrophysiological measures across the three tasks, with the tones used as the neutral condition against which syllables and words were compared. In summarizing the most relevant results, the amplitude of the earlier posterior negativity component (EPN) was larger for extroverted than for introverted participants in the comparison between the emotional conditions and the control one. This effect was demonstrated in parietal electrodes (P3, Pz, P4). Also, a larger P300 was reported for tones than for words, whereas the LPC was larger for words than for tones or syllables. The difference in the study of Jaspers-Fayer et al. (2012) was the combined use of EEG and fMRI measures and the exclusion of the personality trait test. The ERP results of the latter study, reported exclusively for the Pz electrode, showed similar effects on the EPN only.

In our study, we looked at the modulation of the LPC component. Specifically, we examined the cerebral activity of Spanish native listeners when listening to Spanish words that had positive, negative, or neutral connotations and when those words were produced with a native or foreign accent. Our first aim was to replicate the effect of emotional word processing on the LPC component, found in numerous previous studies that had compared single nouns in the visual modality (e.g., Frühholz et al., 2011; Kissler and Herbert, 2012). This was deemed a necessary prerequisite, as our study was the first study in the emotional language literature to look at foreign accent effects. Second, we sought to explore whether the depth of word processing and emotional reaction to words with affective connotations might be influenced by whether those words were produced in a native versus a foreign accent.

## Method

### Participants

Forty right-handed Spanish native speakers, (17 female; *M* = 23 years, *SD* = 0.86), students at Universitat Pompeu Fabra (UPF), took part in the study for financial compensation. Participants were recruited from the database of the *Centre for Brain and Cognition* of UPF and from advertisements displayed on the Centre's Neuroscience Laboratory website (http://cbclab.upf.edu/?q=es/node/25). They all had normal hearing and no record of neurological diseases. Ethical approval was issued by UPF and informed written consent was obtained from all volunteers. Along with the written consent form, participants were also given to complete a detailed language experience questionnaire prior to the experiment to make sure that they fitted the profile of Spanish native speakers who were born and raised in monolingual Spanish families, had little knowledge of any other language, and used Spanish in their daily interactions.

### Materials

Ninety-six experimental items (32 positive, 32 negative, 32 neutral words) and one-hundred four filler words were selected from the *Spanish adaptation of the Affective Norms for English Words* (Redondo et al., 2007) and from *BuscaPalabras* (*B-Pal*; Davis and Perea, 2005). Each word, including filler items, was heard from the same speaker three times in separate blocks to increase statistical power. The first round of presentation was completed after 200 trials, followed by another 400 randomized trials for the second and third repetition, respectively. Thus, a total of 600 trials (288 of which were experimental items) comprised each session. The selected affect-laden words differed in emotional valence [*F*_(2, 93)_ = 837.24, *p* = 0.001, η^2^_*p*_ = 0.947], with positive words (*M* = 7.8; *SD* = 0.60) being rated more pleasant than negative (*M* = 2.0; *SD* = 0.59) or neutral words (*M* = 5.0; *SD* = 0.48); negative words being rated more unpleasant than positive or neutral words; and neutral words being rated more neutral than positive or negative words (all *p*s < 0.05)^1^. Positive and negative words were matched in terms of arousal^2^ (both *M* = 6.2; positive *SD* = 0.89 and negative *SD* = 0.88) but differed from neutral words (*M* = 4.8; *SD* = 0.59); [*F*_(2, 93)_ = 34.16, *p* = 0.001, η^2^_*p*_ = 0.423]. The experimental items were also matched for the following lexical properties^3^: grammatical category (all being nouns); number of syllables; phoneme length; phonological neighbors; word frequency; and mean token bigram frequency: all *p*s > 0.05. (A list of the Spanish experimental stimuli with their English translations is provided in the Supplementary Material).

All stimuli were recorded digitally into 16-bit stereo at 44,100 Hz and their noise removal and normalization were performed using the audio editor software *Audacity 2.0.2*. The manipulation of accent was between participants, because the present study used single words as experimental stimuli which allow less information of foreign-accented speech to unfold and hold on by its listeners. Hence, a within-participant design with single words might jeopardize our aim to obtain clear effects of native-biased or foreign-biased accented speech. We thus used four native speakers of standard Spanish (two males and two females; mean age 26 years) for the listeners of the native accent group, and four speakers of different nationalities (two males and two females: French, Dutch, Hungarian, and German; mean age 28 years) for the listeners of the foreign accent group. The speakers were either postgraduate students or postdoctoral researchers in the *Center for Brain and Cognition* of Universitat Pompeu Fabra. To obtain information on the foreign-accented speakers' knowledge and use of the target language, we asked them to complete a language experience questionnaire, where among others, they provided ratings on their reading and listening comprehension, as well as written and oral production skill in Spanish on a 7-point scale (1 = skill lacking, 7 = as good as native language): French speaker *M* = 4.7, *SD* = 0.95; Dutch speaker *M* = 1.5, *SD* = 0.57; Hungarian speaker *M* = 1.5, *SD* = 0.57; German speaker *M* = 3.25, *SD* = 0.50. At the time of testing, the French speaker had been living in Spain for 3 years, the Dutch speaker for 6 months, and the Hungarian and German speakers for 4 months. All four speakers reported English as their second language (i.e., a language, other than their native language, they had learnt and could hold a fluent conversation in). English was also the language all speakers used most in their daily interactions, with the French speaker also using Spanish.

Four lists were created for the recordings of each accent group. The order of word presentation within and across lists was randomized. Each of the native- and foreign-accented speakers produced all the intended stimuli, so that across participants all words were heard from all the speakers. All speakers were instructed to pronounce each word in neutral vocal expressiveness, as our intention was to examine the effects of accented speech on emotional word processing without affective prosody playing a role (cf. Paulmann and Kotz, 2008). To make sure that the foreign-accented speakers would stress words in the correct way, we asked them to repeat each word that was first produced by a native Spanish speaker. The pace of reading was steady throughout the recording. The mean duration for native-accented words was: positive words *M* = 588 ms; negative words *M* = 596 ms; and neutral words *M* = 534 ms. The mean duration for foreign-accented words was: positive words *M* = 670 ms; negative words *M* = 687 ms; and neutral words *M* = 626 ms.

Foreign-accentedness of the speakers was confirmed by a Spanish native speaker, who also checked the recorded materials for comprehensibility. In addition, another four Spanish native speakers (one male and three females; mean age 30 years) rated all the stimuli for foreign accentedness. Specifically, the raters were asked through on-screen instructions to rate on a 7-point scale how foreign-accented (i.e., not native-like) each word sounded (1 = native-like—7 = foreign-accented). The session was the same as that used for the ERP session, with the exception that each stimulus was heard only once and that headphones were used instead of loudspeakers. Number stickers indicating the rating scale were placed on a keyboard and raters were advised to listen well without delaying their responses. Analyses of Variance between positive, negative and neutral words, treating participants as within-factor and items as between-factor indicated no significant differences in accent across the three conditions: positive *M* = 3.5, *SD* = 1.00; negative *M* = 3.7, *SD* = 0.95; neutral *M* = 3.7, *SD* = 0.95; [*F*1_(2, 6)_ = 1.00, *p* = 0.422, η^2^_*p*_ = 0.250 and *F*2_(2, 93)_ = 1.16, *p* = 0.316, η^2^_*p*_ = 0.025].

### Procedure

Spanish native listeners were randomly assigned to a group of *native accent* (listening to the recordings of native-accented speech) or *foreign accent* (listening to the recordings of foreign-accented speech). Participants were asked to perform a semantic categorization task which was chosen for two reasons: first, to prevent participants from realizing the experimental manipulation regarding the valence of the words, and second and more importantly, to force listeners into deep processing of the stimuli. The latter reasoning was based on findings showing that the LPC can be modulated by the depth of word processing required by the task at hand, such that it may be less enhanced or even absent in superficial tasks that do not require lexico-semantic analysis as opposed to deep processing tasks that do (e.g., Schacht and Sommer, 2009; Hinojosa et al., 2010). The experiment was run with E-prime and the participants' cerebral activity was continuously recorded using Brain Vision Analyzer.

Following the positioning of the electroencephalographic (EEG) cap, participants were given onscreen instructions about the experimental task. They were told that they would hear Spanish words and that their task was to listen carefully to each word and press a button on a keyboard (positioned on their lap) with their left index, if they thought the word referred to something concrete, “something they could touch,” and another button with their right index, if they thought the word referred to something abstract, “something they could not touch.” The order of index response was counterbalanced across participants. Participants were also told that there were not correct or incorrect responses, and that if they were not sure about the status of a word, they should provide a response based on what they thought was most likely the case. They were encouraged to listen to the words well before responding, but to not delay their responses. A short practice session with four items, not used later on, preceded the actual experiment. In each trial a fixation point was presented on a computer screen for 500 ms, followed by a blank screen for another 500 ms, and then by the stimulus which was heard from loudspeakers located on each side of the computer. After 3000 ms, within which participants were expected to have given their responses, a blank screen was displayed for 500 ms until the fixation point appeared after a variable interval of 25, 50, 75, and 100 ms. Five short breaks were allowed throughout the experiment. Each experimental session lasted approximately 40 min.

When debriefed at the end of the experiment, all listeners of the foreign accent group identified that the speakers had a foreign accent (i.e., that the speakers were non-native speakers of the target language). Subsequently, when participants were asked to identify the native language background of the speakers, the majority preferred to answer that they were “foreign.” Of those participants who made an attempted guess, no single accent was identified accurately by all the guessers, suggesting the listeners' lack of familiarity with the foreign accents used. Next, participants were asked to provide an estimate of the words they were unable to understand due to the speakers' foreign accent: in a total of 600 words (including fillers and three repetitions of each word), out of the 18 participants who were the listeners of the foreign accent group, 7 participants reported not having understood 1 word; 1 participant reported not having understood 1–2 words; 1 participant reported not having understood 2 words; 1 participant reported not having understood 3 words; and 3 participants reported not having understood 2–3 words. Thus, listeners' responses did not suggest any processing bias or difficulty toward a particular foreign accent, since no single accent was picked up by the majority of the participants. Importantly, responses demonstrated that, although participants were able to perceive foreign accentedness in speakers' speech and although they did not have familiarity with the speakers' accents, foreign accent strength was moderate since it did not affect the words' comprehensibility.

#### Electrophysiological recording and analysis

Electrophysiological data were recorded continuously at a sampling rate of 500 Hz from 32 tin electrodes placed according to the 10–20 convention, in reference to an electrode placed on the participant's nose tip and re-referenced to the average of linked mastoids offline. Impedance was kept below 5 kΩ. Eye movements were monitored by two external electrodes placed horizontally (outer canthus) and vertically of the right eye. EEG data were filtered off-line to 0.1 Hz high-pass filter and 30 Hz low-pass filter (cf. Luck, 2005 for recommendation on the use of filters in ERP studies and Goslin et al., 2012 for a similar cut-off procedure used in a related study). Eye blink artifacts were mathematically corrected using Gratton et al.'s procedure (1983), implemented in *Brain Vision Analyzer 2.0, Brain Products*. Epochs ranged from -100 to 1200 ms and segments containing artifacts (brain activity above or below 100 μV or a change in amplitude between adjacent segments of more than 200 μV) or eye blinks were excluded. Baseline correction was performed in reference to −100 ms pre-stimulus activity. Due to excessive artifacts (fewer than 60% of trials in each condition), three participants were excluded from the analysis, leading to a final analysis of 37 participants: 19 in the *native accent* group and 18 in the *foreign accent* group. The mean number of segments that was calculated in each condition per participant group was as follows: *native accent*; positive 90, negative 90, neutral 89, and *foreign accent;* positive 87, negative 87, neutral 85.

In line with previous studies from the emotional language literature (e.g., Kanske and Kotz, 2007; Frühholz et al., 2011) and upon visual inspection of the Grand Average for each condition related to our research question, we selected the LPC component for analysis. We then broke its analysis into two time-windows of 600–950 and 950–1200 ms to prevent the long lasting positivity from smoothing out any effects. Despite our focus being on the LPC component, we also looked at the N400 for earlier effects of lexical-semantic processing (cf. Wambacq and Jerger, 2004; Goslin et al., 2012; Hanulíková et al., 2012) in the 300–550 ms time-window.

The effect of emotion was broadly distributed, being particularly prominent over posterior scalp sites consistent with previous studies from the relevant literature (Kotz and Paulmann, 2011; Citron, 2012). To obtain a fine-grained picture of the distribution of the effects, since the effect seemed to be larger in posterior electrodes than in centro-parietal electrodes, we selected the centro-parietal (CP) and parieto-occipital (PO) regions for analysis. Mean amplitudes (average of the ERP amplitude measure in a given interval) measured in μV for the LPC component and each participant were submitted to a mixed ANOVA design. Region (*CP* vs. *PO*), Electrode (CP: *C3, Cz, C4, CP1, CP2*—PO: *P3, Pz, P4, PO1, PO2*), and Valence Type (*positive* vs. *negative* vs. *neutral*) were used as within-participant factors and Accent Group (*native* vs. *foreign*) as between-participant factor. A 0.05 level of significance was used. In what follows, we report significant main effects that are relevant to our research question and significant interactions that include the manipulated variables. Bonferroni correction was used for multiple comparisons and Greenhouse-Geisser corrected values are reported throughout.

## Results

### Behavioral performance

In a total of 10656 trials, 188 (1.8%) trials were excluded from the analysis as outliers, using a ±2.0 as exclusion criterion and being below or above the response time limit (200-2500 ms). Another 160 (1.5%) trials were discarded due to response recording failure. The remaining latencies, all measured from voice onset, were analyzed in a Valence Type (3) × Accent Group (2) mixed ANOVA design, with the first factor treated as within-participants and the second factor treated as between-participants. The results yielded a significant main effect of Valence Type [*F*_(2, 70)_ = 26.65, *p* = 0.001, η^2^_*p*_ = 0.432], with emotional words responded to slower than neutral words. Pairwise comparisons indicated that positive and negative words were equally slow (*M* = 1249 ms, *SE* = 22.85/*M* = 1252 ms, *SE* = 22.99; *p*s > 0.05) when compared to neutral words (*M* = 1177 ms, *SE* = 23.66; *p* = 0.001). The effect of Accent Group was not significant: Native accent group; *M* = 1205 ms, *SE* = 30.91; Foreign accent group; *M* = 1247 ms, *SE* = 31.75; [*F*_(1, 35)_ = 0.93, *p* = 0.341, η^2^_*p*_ = 0.026], nor was the two-way interaction [*F*_(2, 70)_ = 1.04, *p* = 0.359, η^2^_*p*_ = 0.029].

Because during the selection of the stimuli it was not possible to also control for concreteness, and because it has been suggested that emotion and concreteness effects are intertwined (e.g., Kousta et al., 2009, 2011; Vigliocco et al., 2013), we conducted further analyses taking into account tactile perception of the stimuli as the only objective measure closest to concreteness (but see Connell and Lynott, 2012). Hence we split latencies considering participants' distribution of responses across “touchable” and “non-touchable,” with Tactile Perception, Valence Type, and Accent Group as factors. The results yielded a significant main effect of Tactile Perception, [*F*_(1, 35)_ = 32.34, *p* = 0.001, η^2^_*p*_ = 0.480], with words considered “touchable” responded to faster than words considered as “non-touchable”: *M* = 1216 ms, *SE* = 23.01 vs. *M* = 1313 ms, *SE* = 27.63. There was also a significant interaction between Valence Type and Tactile Perception, [*F*_(2, 70)_ = 66.27, *p* = 0.001, η^2^_*p*_ = 0.654], showing that for “touchable” responses, neutral words were faster than negative and positive words, and negative words were faster than positive words: all *p*s < 0.05. For “non-touchable” responses, positive words were faster than negative and neutral words, and negative words were faster than neutral words: all *p*s < 0.05. No effects that involved the factor Accent Group were significant. (Table 1 presents overall response latencies and latencies distributed across “touchable” and “non-touchable” responses).

**Table 1:** **Response latencies in ms and standard errors in brackets of participants' performance on the semantic categorization task**.

**Accent group**	**Semantic categorization**	**Valence type**		
		**Positive**	**Negative**	**Neutral**
Native	**Overall**	**1224** (31.9)	**1225** (32.1)	**1165** (33.0)
	Touchable	1271 (38.5)	1203 (35.1)	1136 (31.2)
	Non-touchable	1209 (33.9)	1261 (35.8)	1407 (55.9)
Foreign	**Overall**	**1274** (32.8)	**1280** (32.9)	**1188** (33.9)
	Touchable	1313 (39.6)	1220 (36.1)	1154 (32.1)
	Non-touchable	1260 (35.0)	1316 (36.8)	1426 (57.5)

**Figure 1:**
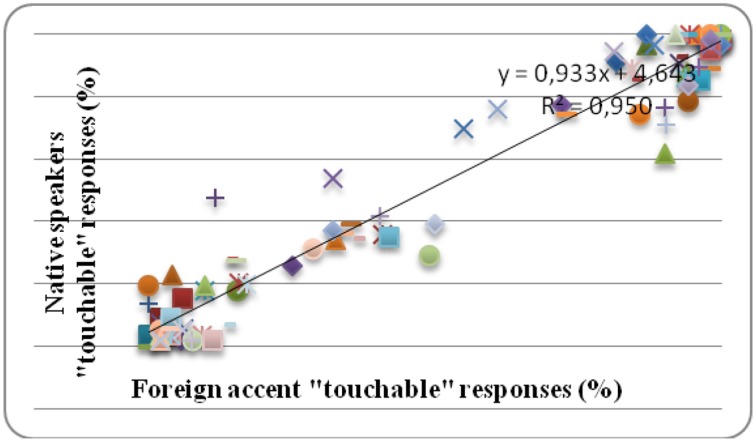
**Percentage (%) of categorical responses for each word of each valence type in each accent group**.

Moreover, we examined the internal consistency of responses to compare the groups' performance for each stimulus at a conceptual—perceptual level. As it is clear in Figure 1, both groups' responses were all in the same direction. That is, a word that was considered “touchable” by the native accent group was also considered “touchable” by the foreign accent group and vice versa. Hence consistency of responses suggested similar conceptual and perceptual categorization across the two groups.

### Electrophysiological performance

#### Late positive complex: 600–950ms time window—main analysis^4^

**Figure 2:**
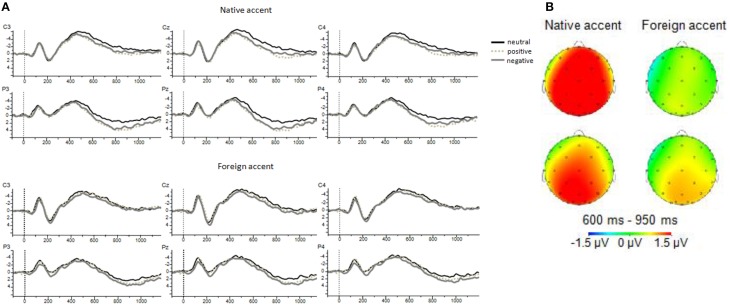
**(A)** ERP waveforms of representative electrodes included in the analysis of the three experimental conditions across the two accent groups: solid black line for neutral words; dashed gray line for positive words; and solid gray line for negative words. Negative is plotted up. **(B)** Topographic maps of the wave difference of positive word amplitudes minus neutral word amplitudes (top panel); and negative word amplitudes minus neutral word amplitudes (bottom panel) in each accent group.

The results of the analysis on the LPC component yielded a significant main effect of Valence Type, with positive and negative words showing a larger amplitude than neutral words (*p*s < 0.05). The difference between the two groups of emotional words was not significant (*p* > 0.05); positive words: *M* = 0.71 (MSE = 0.48) and negative words: *M* = 0.69 (MSE = 0.53). There was also a significant three-way interaction between Region, Electrode, and Valence Type, showing that the Electrode × Valence Type interaction was significant in the centro-parietal region [*F*_(3.95, 142.31)_ = 4.27, *p* = 0.003, η^2^_*p*_ = 0.106], but not in the parieto-occipital region [*F*_(4.57, 164.71)_ = 0.27, *p* = 0.916, η^2^_*p*_ = 0.007]. Crucially, the interaction between Valence Type and Accent Group was significant, with the Valence Type effect being significant in the native accent group for both positive and negative words: *t*_(18)_ = 4.80, *p* = 0.001 and *t*_(18)_ = 3.09, *p* = 0.006, respectively. For the listeners of the foreign accent group the effect of Valence Type was significant only in the comparison between negative and neutral words: *t*_(17)_ = 2.17, *p* = 0.044. (Table 2 reports the results of the analysis of the LPC component in the 600–950 ms time window. Figure 2A shows the ERP waveforms for emotional and neutral words and Figure 2B the topography of the effects.)

**Table 2:** **Results of the analysis of the Region (2) × Electrode (5) × Valence Type (3) × Accent Group (2) mixed ANOVA in the 600–950 ms time window of the LPC component**.

**Independent Variable**	**df**	**F**	**p**	**η^2^_*p*_**
Region	(1, 35)	112.98	0.000	0.763
Electrode	(2.44, 85.45)	36.12	0.000	0.508
**Valence Type (VT)**	(2, 70)	12.29	**0.000**	0.260
**Accent Group (AG)**	(1, 35)	1.11	**0.299**	0.031
Region × Electrode	(2.52, 88.43)	11.38	0.000	0.245
Region × VT	(2, 70)	1.48	0.233	0.041
Region × AG	(1,35)	0.26	0.613	0.007
Electrode × VT	(3.95, 138.41)	2.08	0.087	0.056
Electrode × AG	(2.44, 85.45)	0.44	0.681	0.013
**VT × AG**	(1.81, 63.55)	3.47	**0.041**	0.090
Region × Electrode × VT	(4.83, 169.06)	3.54	0.005	0.092
Region × Electrode × AG	(2.52, 88.43)	1.51	0.222	0.041
Region × VT × AG	(1.93, 67.79)	0.06	0.934	0.002
Electrode × VT × AG	(3.95, 138.41)	0.46	0.759	0.013
Region × Electrode × VT × AG	(4.83, 169.06)	0.76	0.576	0.021

**Figure 3:**
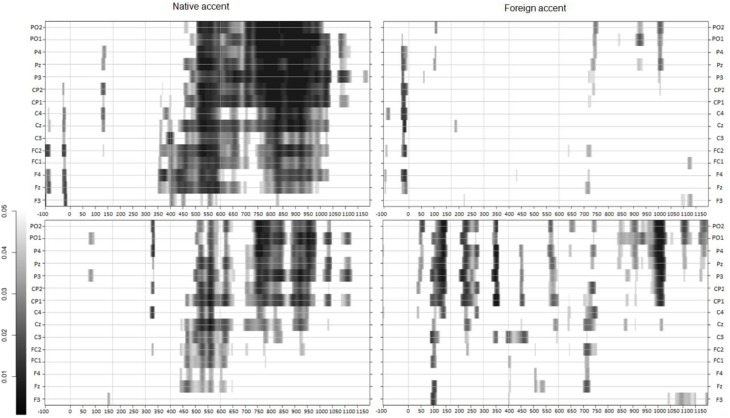
**Paired *t*-tests performed at each sampling point (every 2 ms) in each accent group. Top panel:** positive vs. neutral words; **bottom panel:** negative vs. neutral words. Colored points correspond to *p*-values below 0.05, with darker shades showing larger significance. Electrodes from the frontocentral region are also included for comparison purposes.

To better capture the evolution of the valence type effect in each accent group, we ran a two-tailed paired *t*-test at every sampling point (2 ms) and in each electrode. Then, we contrasted positive and neutral conditions and negative and neutral conditions. In accord with the effects displayed on the topographic maps in Figures 2B, 3 clearly shows that the effects of emotional word processing were more prominent for native-accented speech than for foreign-accented speech, especially when positive words were involved, and that the effects started earlier in the native than in the foreign accent^5^.

#### Late positive complex: 600–950ms time window—restricted analysis on “touchable” responses

The manipulation of accented speech yielded modulated effects in the processing of emotional words in the 600–950 ms time window. Yet because it was unclear whether the observed effects were the result of processing tactile perceptual features of the words rather than of their emotional content, we conducted additional analyses to capture a finer picture of the suggested effects. We thus considered ERPs that were yielded for “touchable” responses only, so that any differences found between emotional and neutral words would be attributed to the processing of affective-laden information. A total of 2730 segments (1272 emotional—1458 neutral), that corresponded to “touchable” words, were used for the native accent group, and 2451 (1109 emotional—1342 neutral) were used for the foreign accent group.

The analysis yielded a marginally significant main effect of Valence Type [*F*_(2, 70)_ = 2.94, *p* = 0.056, η^2^_*p*_ = 0.078] and a significant Valence Type x Accent Group interaction [*F*_(1.96, 68.83)_ = 3.72, *p* = 0.030, η^2^_*p*_ = 0.096]. Follow-up analyses showed a valence type effect in the native accent group, with a larger amplitude for positive than for neutral words: *M* = 0.92 (MSE = 0.81) vs. *M* = –0.03 (MSE = 0.58), respectively; *t*_(18)_ = 2.14, *p* = 0.047. In the foreign accent group, the only significant difference was between negative and positive words, with a larger amplitude for negative than for positive words: *M* = 0.39 (MSE = 0.83) vs. *M* = –1.18 (MSE = 0.76), respectively; *t*_(17)_ = 3.74, *p* = 0.002. Although we had no a priori prediction regarding the latter finding, it might be attributed to negative processing bias, (cf. Kanske and Kotz, 2007), whereby heightened attention to negative words rendered their processing resistant to the effects of foreign-accented speech. Taken together, despite a smaller number of segments used in the *post-hoc* restricted analysis, the effect that was demonstrated differed across the two accent groups. Hence the observed effects in the main analysis could not have been driven by the haptic-perceptual features of the words (some being less touchable than others), but partly reflected emotion modulation; the culprit of this modulation being foreign-accented speech. We defer to the Discussion on this issue in more detail.

#### Late positive complex: 950–1200ms time window

Analyses in the 950–1200 ms time window yielded a significant main effect of Valence Type, with positive and negative words showing a larger amplitude than neutral words: *M* = 1.13 (MSE = 0.45); *p* = 0.05 and M = 1.28 (MSE = 0.45) vs. M = 0.53 (MSE = 0.35); *p* < 0.05, respectively. The difference between the two groups of emotional words was not significant (*p* > 0.05). Finally, there was a significant interaction between Region and Valence Type, showing that the effect of Valence Type was significant in the parieto-occipital region [*F*_(2, 72)_ = 8.03, *p* = 0.001, η^2^_*p*_ = 0.182], but not in the centro-parietal region [*F*_(2, 72)_ = 2.45, *p* = 0.093, η^2^_*p*_ = 0.064]. As it is reported in Table 3, the effect of valence type did not interact with accent group.

**Table 3:** **Results of the analysis of the Region (2) × Electrode (5) × Valence Type (3) × Accent Group (2) mixed ANOVA in the 950–1200 ms time window of the LPC component**.

**Independent variable**	**Df**	**F**	**p**	**η^2^_*p*_**
Region	(1, 35)	41.87	0.000	0.545
Electrode	(2.35, 82.31)	12.18	0.000	0.258
**Valence Type (VT)**	(2, 70)	5.41	**0.007**	0.134
**Accent Group (AG)**	(1, 35)	0.01	**0.941**	0.000
Region × Electrode	(3.03, 106.19)	20.00	0.000	0.364
Region × VT	(1.54, 53.99)	6.63	0.005	0.159
Region × AG	(1, 35)	0.11	0.741	0.003
Electrode × VT	(3.23, 113.20)	1.31	0.272	0.036
Electrode × AG	(2.35, 82.31)	0.18	0.866	0.005
**VT × AG**	(1.95, 68.48)	1.51	**0.228**	0.041
Region × Electrode × VT	(4.22, 147.86)	2.08	0.082	0.056
Region × Electrode × AG	(3.03, 106.19)	1.90	0.133	0.052
Region × VT × AG	(1.54, 53.99)	2.00	0.155	0.054
Electrode × VT × AG	(3.23, 113.20)	1.26	0.290	0.035
Region × Electrode × VT × AG	(4.22, 147.86)	0.72	0.581	0.020

## Discussion

Drawing on evidence from sociolinguistic and psycholinguistic studies (e.g., Fuertes et al., 2012; Hanulíková et al., 2012; Pantos and Perkins, 2013), showing effects of foreign-accented speech in various social, cognitive, and linguistic contexts, the present study looked at the interesting perspective of emotional word processing. In particular, it explored whether and how basic linguistic processes related to affective processing and emotion (e.g., Lang, 1979; Bower, 1981) might be modulated by foreign accent. Thus, it examined the case where the meaning of an emotional word's referent was maintained (cf. Hanulíková et al., 2012), but there was a difference between stored phonological representations of a native variety and their phonological variants of foreign-accented speech.

To explore the effects of accented speech in emotional word processing, we used Event Related Potentials (ERPs) and recorded the cerebral activity of Spanish native listeners who performed a semantic categorization task on emotional and neutral words produced with a native or foreign accent. The behavioral results showed that listeners from both accent groups responded slower to emotional words than to neutral words. Response delay for negative words has been reported before (Algom et al., 2004; Estes and Verges, 2008) and has been explained in attention-grabbing terms, when the behavioral task does not require explicit valence-related responses. However, a number of other studies, which have used in their majority lexical decision or affective evaluation tasks, have reported facilitatory effects for emotional words (e.g., Kousta et al., 2009; Schacht and Sommer, 2009), while others have showed a processing advantage for positive over negative and/or neutral words (e.g., Kuchinke et al., 2005; Kanske and Kotz, 2007, 2011). The overall slowdown for both negative and positive words in the present study is most likely associated with the nature of the behavioral task we employed: emotional words required more time than neutral words to be identified as “touchable” (Paivio, 1986; but see Hinojosa et al., 2014 who used the same categorization task as the present study but did not find differences in latencies between emotional and neutral words). Looking at “non-touchable” responses, analyses showed that emotional words were responded to faster than neutral words. Finally, the distribution of responses into “touchable” and “non-touchable” showed that the responses of both listener groups were consistent, and that both groups performed similarly. This is important because despite similar behavioral performance there were still ERP differences across the two accent groups.

The electrophysiological results suggested that the processing of aspects of words related to affective connotations mainly took place in the time window of the Late Positive Complex (LPC) component. This was not surprising, as weak or absent emotion effects on the N400 component is common when the meaning of emotional words is processed in isolation, or in tasks that do not encourage emotional anticipation (Kissler et al., 2006; cf. Kutas and Federmeier, 2000). The LPC, associated with emotional language processing (see Kotz and Paulmann, 2011 for a review), yielded a long lasting effect in response to valence manipulation. This effect was predominant over posterior scalp sites replicating previous ERP studies that have examined the effects of noun affective valence (e.g., Hinojosa et al., 2014; see Citron, 2012 for a review). Crucially, the interaction between valence type and accent group that was yielded in the 600–950 ms time window suggested differential processing across the two groups at an electrophysiological level. Namely, the two accent groups clearly differed in their response to positive versus neutral words, but yielded an effect in the same direction in the comparison between negative and neutral words. The observation of these differences was also supported by the additional paired *t*-tests we conducted every 2 ms to examine the evolution of the valence type effect in each accent group. This analysis was revealing in that it showed that processing of affective aspects of words was susceptible to the effects of accented speech, with more robust and earlier effects in native than in foreign accent, especially for positive words.

The difference in processing positive and negative valence words by the foreign accent group can be entertained by at least two accounts. The first one would support that foreign-accented speech might induce violation of expectations for positive words, assuming that foreign accent in the present study was received as negatively valenced^6^. This could be perceived as incongruence between prosody and semantics, and lead to differential processing of positive words. However, this account would primarily predict robust effects on the N400 or other N400-like component (cf. Bostanov and Kotchoubey, 2004; Wambacq and Jerger, 2004; Paulmann and Kotz, 2008), which was not what we found in the present study. Also, the current experimental context (single word processing) did not create any anticipation as to the semantics of any given stimulus so as to assume that this anticipation in positive word processing was then violated. The second account perhaps more convincingly would argue that negative valence words which typically elicit heightened attention (e.g., Lang et al., 1997; Hinojosa et al., 2014) were more resistant to foreign accent. Hence, we could claim that attentional vigilance to this type of words was above and beyond any effects of foreign-accented speech. No matter which of the above interpretations we accept, a thorny question that may follow is why the behavioral results do not support fully any of these accounts. At present, we cannot readily provide any solid answer other than that the behavioral task we used might not have been sensitive enough to reflect all these differences. Other related studies have also showed dissociation between behavioral and electrophysiological effects (Begleiter et al., 1979; Kanske and Kotz, 2007; Barber et al., 2013; Hinojosa et al., 2014), suggesting that behavioral processes accompanying judgment tasks do not always strictly translate into the observed electrophysiological effects.

The effect of valence and its interaction with accent in the present study was clearly demonstrated on the LPC component, where elaborate processing of emotional aspects of words, but also of concreteness, is assumed to take place (e.g., Kanske and Kotz, 2007; Schacht and Sommer, 2009). Despite the fact that the additional restricted analysis we conducted to address the limitation of controlling for concreteness also yielded processing differences between native and foreign-accented speech, the current data do not allow identifying the extent to which haptic characteristics related to concreteness might have contributed to the observed effects (see also Connell and Lynott, 2012; Hinojosa et al., 2014). Thus, a reasonable assumption would be to interpret our findings considering conjoint effects, an assumption that is compatible with the findings of other relevant behavioral, electrophysiological, and neuroimaging studies (e.g., Kanske and Kotz, 2007; Kousta et al., 2009, 2011; Skipper and Olson, 2014). What these studies suggest is that both valence and variables related to concreteness contribute to emotional word processing; both have a temporal overlap; and both share certain processes which activate overlapping brain areas.

To sum up, the present study replicated ERP effects in emotional word processing of visual word recognition and extended them to spoken word recognition. More interestingly, it showed that aspects of words that are related to affective connotations can be modulated by accented speech, with negative valence resisting these effects. On the basis of the current findings that access to emotional connotative information is not independent of a speaker's accent, one could speculate that foreign accent might also affect other aspects of elaborate word processing, emotion-related information just being one of them. As such, we do not refute an account of effects of foreign-accented speech for other higher order semantic processes. Since in the present study we manipulated affective valence and replicated previously reported effects on the LPC component (e.g., Schacht and Sommer, 2009; Frühholz et al., 2011; Kissler and Herbert, 2012), we discuss the present findings in light of the emotional language literature.

Modulation of emotion effects so far has been reported for emotional content processing in a second language (e.g., Conrad et al., 2011; Opitz and Degner, 2012; Costa et al., 2014). Attenuated effects of emotion through emotional distance and detachment in second language processing have been attributed to subjective experience related to age of acquisition and proficiency in a second language, or to the cognitive load imposed by processing a second language (e.g., Pavlenko, 2005; see Caldwell-Harris, 2014 for a recent review). However, the present study was conducted in participants' native language. The listeners of foreign accent were also native speakers of the target language and during the experiment listened to words they themselves were familiar with and had often used in their daily interactions. And yet, they showed differential neural effects in comparison to the group of native accent. As the current study was exploratory and was not designed to identify specific foreign accent characteristics or test any specific theory of emotional language processing, in what follows we provide tentative explanations that merit further investigation.

The present study answered the questions *whether* and *how* processing words with affective connotations in a listener's native language may be influenced by accented speech. One way to answer *why* foreign accent might modulate affective-laden information processing is to assume that emotional word processing extends beyond the level of mere word recognition. During spoken word processing, listeners do not only retrieve from their memory linguistic information stored about words, but also extra-linguistic information retained about how words sound when produced by a speaker (Palmeri et al., 1993; Nygaard et al., 1994; Goldinger, 1996, 1998; Johnson, 1997; Pisoni, 1997). Word recognition is achieved upon successful matching of properties of a given speech signal to stored exemplars of similar acoustic features, which leads to activation of relevant linguistic information (e.g., grammatical and semantic information; see Weber and Scharenborg, 2012 for a review of models of spoken word recognition). When considering the processing of affective-laden words, we may additionally assume the contribution of operations of elaborate word processing that involve aspects of emotional significance retrieved from episodic representations (Phelps, 2004). If those operations are not adequately performed due to the effects of foreign accent, then differences between native and foreign-accented speech might occur. In other words, resources dedicated to extracting episodic information might be distracted by processing foreign-accented cues and not fully engage with processing emotionally loaded content. Thus, we suggest that in the present study foreign accent exerted its effects at a stage of semantic and affective-connotation processing where emotionally-loaded content was under way. This explanation might also account for the effect of foreign accent on the early window of the LPC component, suggesting that emotional significance had not fully kicked in yet. Clearly, we also assume that regular exposure to the same phonological variants would gradually lead to restructuring listeners' phonological representations (Bradlow and Bent, 2008; Kraljic et al., 2008). Thus, variability would eventually be accommodated in the speech signal, enhance cost-free, deep word processing, and ultimately reduce the effects of foreign-accented speech for affective-laden words.

A simpler but perhaps less interesting account might view foreign-accented speech as a particular type of “noise” (see Adank et al., 2012). Under such an account, foreign accent effects on emotional word processing could be attributed to mere perceptual demands, influencing higher order semantic and affective processing. We hope future research will be able to test both these accounts.

## Conclusions

Understanding whether and how basic linguistic processes might be modulated by foreign-accented speech can have important implications for social and interpersonal communication, especially nowadays with demographic changes caused by socioeconomic challenges and global interdependence. Our study showed that foreign-accented speech can impact to some extent on higher order processes relating to emotionally loaded semantic information. By doing so, it shed light on a fascinating but underexplored topic and opened up new avenues of research where other critical questions should be further addressed: Which aspects exactly of foreign-accented speech can modulate emotional word effects? Can affective prosodic cues be used as a crutch to emotional word processing and cancel out foreign accent effects? How are such effects demonstrated at the more complex level of sentence representation? These are only some of the questions in need of further investigation with the potential to impact on a variety of research areas, such as word representation and spoken word recognition, second language learning, and language pathology to name but a few.

### Conflict of interest statement

The authors declare that the research was conducted in the absence of any commercial or financial relationships that could be construed as a potential conflict of interest.

## Footnotes

^1^Ratings of Valence and Arousal are scored on a 9-point Likert scale: For valence 1 = negative, 5 = neutral, 9 = positive; for arousal: 1 = low arousal, 9 = high arousal.

^2^Here and throughout, decimals are rounded to the nearest tenth.

^3^Means and SDs for the lexical properties of the experimental stimuli used in the present study; number of syllables: positive *M* = 3.0, *SD* = 0.73; negative *M* = 3.1, *SD* = 0.83; neutral *M* = 2.7, *SD* = 0.72; phoneme length: positive *M* = 6.9, *SD* = 1.77; negative *M* = 7.1, *SD* = 1.74; neutral *M* = 6.2, *SD* = 1.43; phonological neighbors: positive *M* = 2.0, *SD* = 3.98; negative *M* = 1.2, *SD* = 1.61; neutral *M* = 2.9, *SD* = 3.82; word frequency: positive *M* = 73.9, *SD* = 84.70; negative *M* = 34.4, *SD* = 62.1; neutral *M* = 59.2, *SD* = 78.2; and mean token bigram frequency: positive *M* = 555.9, *SD* = 351.65; negative *M* = 487.0, *SD* = 342.73; neutral *M* = 579.5, *SD* = 302.68.

^4^The results of the analysis on the N400 component in the 300–550 ms time window did not yield any significant effect that involved the manipulated variables of Valence Type and Accent Group or the interaction between them.

^5^Because the t-tests also showed effects in frontocentral electrodes in the N400 time window, which were not included in the main analysis, we considered the electrodes Fz, FC1, and FC2 in an additional analysis. The results showed that the amplitude for neutral words was larger than for positive words in the native accent group *t*_(18)_ = 3.20, *p* = 0.005, but not in the foreign accent group *t*_(17)_ = 0.791, *p* = 0.440. The comparison between neutral and negative words did not reach significance in either group: *t*_(18)_ = 1.86, *p* = 0.079 and *t*_(17)_ = 1.52, *p* = 0.147, respectively.

^6^We thank the reviewer for suggesting this interpretation.
