# Supplementary material for: The way you say it, the way I feel it: emotional word processing in accented speech
Source: Front Psychol. 2015 Mar 27;6:351. doi: 10.3389/fpsyg.2015.00351 (PMC4376116; doi:10.3389/fpsyg.2015.00351)
Supplement: Supplementary file 1 [file DataSheet1.DOCX]

**APPENDIX**

Experimental stimuli used in the study with their English translations in brackets.

**Positive Words Negative Words Neutral Words**

corazón (heart) violación (rape) corredor (runner)

navidad (christmas) muerte (death) pasillo (corridor)

abundancia (abundance) guerra (war) hierro (iron)

milagro (miracle) entierro (burrial) máquina (machine)

ángel (angel) destrucción (destruction) esquina (corner)

justicia (justice) enfermedad (sickness) lluvia (rain)

riqueza (riches) traidor (traitor) carro (wagon)

cachorro (puppy) pobreza (poverty) herramienta (tool)

vencedor (champion) ataúd (coffin) vidrio (glass)

estrella (star) matanza (slaughter) mentón (chin)

conocimiento (knowedge) veneno (venom) camión (truck)

confianza (trust) atrocidad (outrage) lavabo (sink)

juventud (youth) odio (hatred) consigna (locker)

regalo (present) quemadura (burn) tenedor (fork)

pareja (couple) fiebre (fever) chaleco (vest)

maravilla (wonder) cárcel (jail) reloj (clock)

jardín (garden) mentira (lie) edificio (building)

canción (song) ladrón (thief) lápiz (pencil)

esperanza (hope) chantaje (blackmail) carretera (highway)

abrazo (hug) suciedad (dirt) fuego (fire)

familia (family) inundación (flood) armario (cabinet)

madre (mother) amenaza (menace) cordero (lamb)

paraíso (paradise) desventaja (handicap) lámpara (lamp)

fiesta (party) infierno (hell) ordenador (computer)

caricia (caress) ejecución (execution) granja (farm)

diversión (fun) soledad (loneliness) calle (street)

risa (laughter) cuchillo (knife) llave (key)

amigo (friend) mendigo (beggar) tierra (earth)

beso (kiss) cicatriz (scar) ciudad (city)

placer (pleasure) araña (spider) ejercicio (exercise)

amor (love) tiburón (shark) nombre (name)

libertad (freedom) aguja (needle) ventana (window)
